# Ewing Sarcoma Family of Tumors (ESFTs) of Renal Origin Presenting With Bone Metastases: A Case Report

**DOI:** 10.7759/cureus.82829

**Published:** 2025-04-23

**Authors:** Tiffany E Jakowczuk, Gord G Zhu, Jeffrey J Tomaszewski, Tae Won Kim, Marc Zeffren, Daisy C Obiora, Veniamin Barshay, Tina B Edmonston, Hadi Shojaei, Ruth Birbe

**Affiliations:** 1 Cooper Medical School, Rowan University, Camden, USA; 2 Pathology, Cooper University Hospital, Camden, USA; 3 Urology, Cooper University Hospital, Camden, USA; 4 Orthopedics, Cooper University Hospital, Camden, USA; 5 Radiology, Cooper University Hospital, Camden, USA

**Keywords:** ewing sarcoma (es), ewing sarcoma family of tumors(esft), ewing sarcoma of the kidney, extra-skeletal ewing sarcoma, fluorescence in situ hybridization (fish), metastatic renal cancer, next generation sequencing (ngs), res: renal ewing sarcoma, urologic neoplasms

## Abstract

We report a unique case of a female patient in her fifth decade of life who presented with groin pain and was found to have a right kidney mass measuring approximately 8 cm, in addition to multiple bone metastases. Both the nephrectomy specimen and the bone biopsy were found to belong to the Ewing sarcoma family of tumors (ESFTs). This was confirmed by immunohistochemical studies and fluorescence in situ hybridization (FISH) showing *EWSR1* gene rearrangement. Molecular analysis with next-generation sequencing (NGS) showed a type II *EWSR1:FLI1* gene fusion. The patient’s disease progressed rapidly, and she passed away approximately three months after admission. Although extremely rare in the kidney, ESFT should be considered in patients who present with clinically aggressive kidney tumors.

## Introduction

Ewing sarcoma family of tumors (ESFTs), which includes Ewing sarcoma and primitive neuroectodermal tumors (PNETs), is a group of aggressive malignant neoplasms characterized by small round blue cells and fusion of the ​​​​​​*EWSR1* gene with other genes such as *FLI1* and *ERG *[1]. ESFT of renal origin behaves more aggressively than those arising in other sites, including bone [2-6]. Up to 50% of patients present with metastasis, and only 50% of those survive after five years [2-3].

## Case presentation

**Figure 1:**
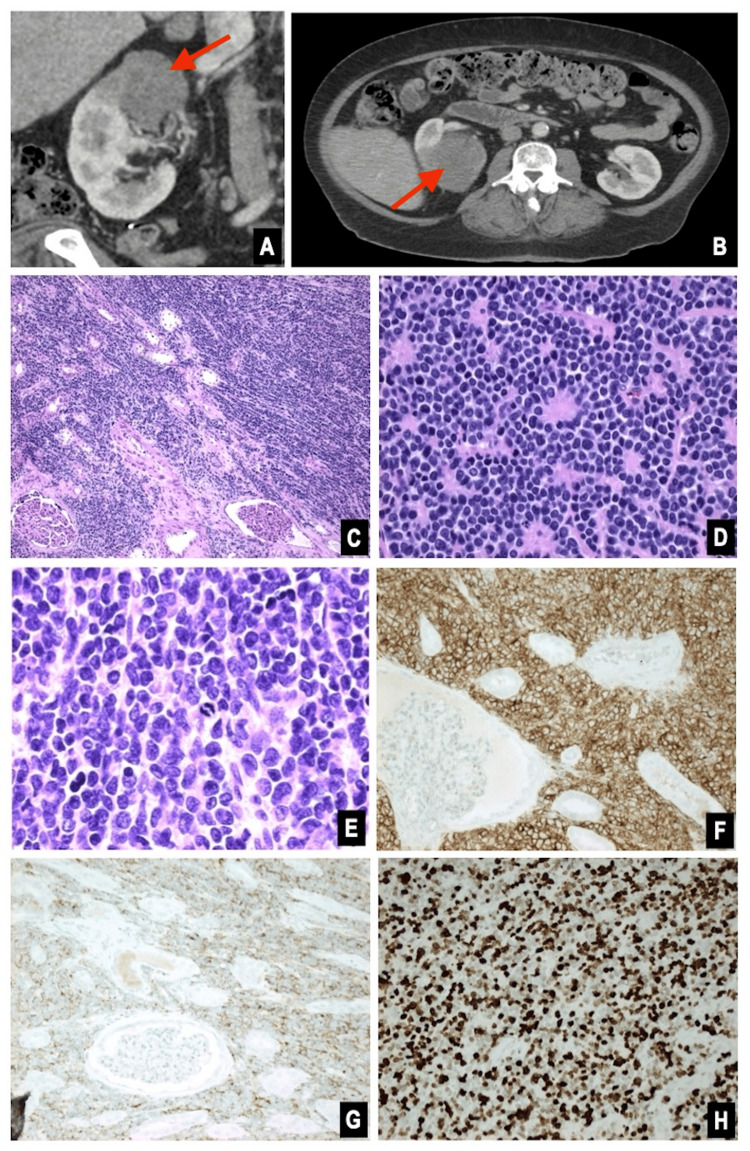
Right renal mass. Computed tomography (CT): coronal (A) and axial (B), with area of interest identified by arrows. Pathology of nephrectomy: hematoxylin and eosin (H&E) stain at 100x (C), 400x (D), and 600x (E). Immunohistochemical stains positive for CD99 (F), synaptophysin (G), and Ki-67 with a high proliferation index between 80% and 90% (H).

**Figure 2:**
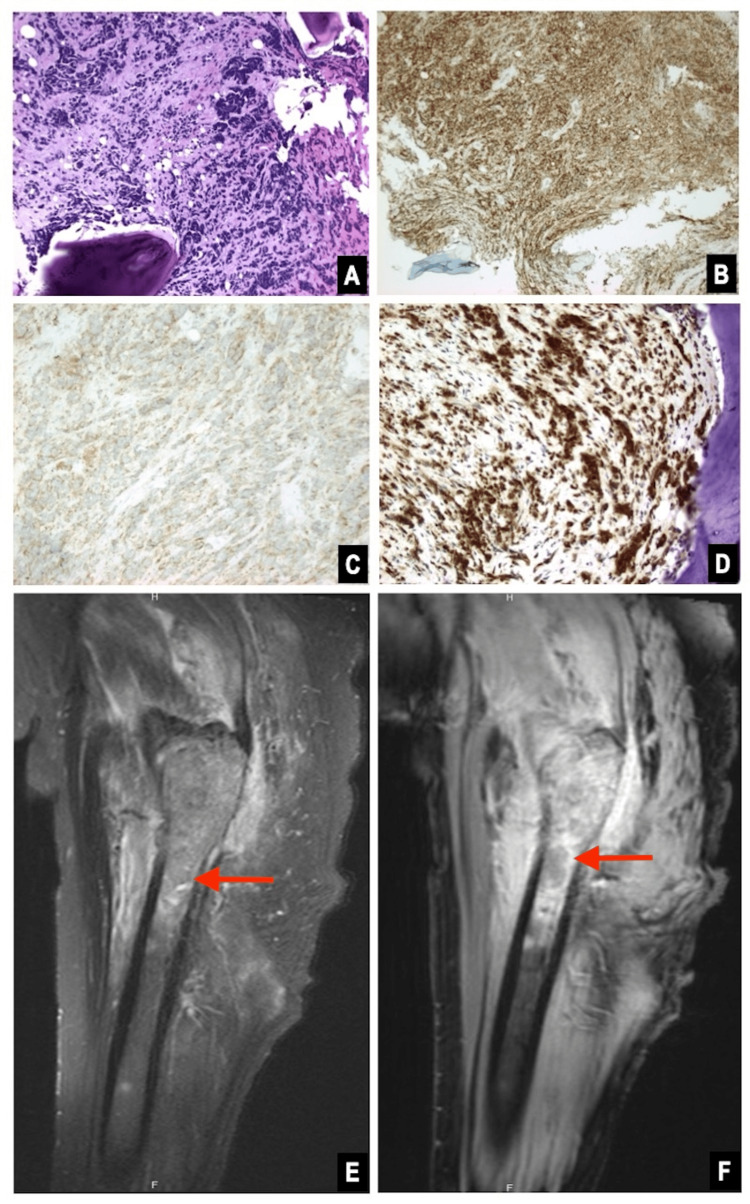
Right femur lesion. Pathology of bone biopsy: hematoxylin and eosin (H&E) at 200x (A). Immunohistochemical stains positive for CD99 (B), synaptophysin (C), and Ki67 with a high proliferation index (D). Magnetic resonance imaging (MRI): T1 fat-saturated before (E) and after (F) contrast, sagittal view, with area of interest identified by arrows.

**Figure 3:**
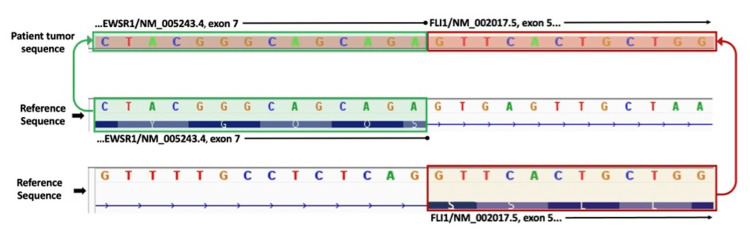
Next-generation sequencing (NGS) of the kidney tumor demonstrating a type II fusion of exon 7 of EWSR1 with exon 5 of FLI1.

A woman in her fifth decade of life with a history of diabetes and nephrolithiasis presented with groin pain. Computed tomography (CT) imaging of the pelvis revealed a 7.8 cm centrally necrotic, hypovascular right renal mass (Figure 1). Magnetic resonance imaging (MRI) and a nuclear bone scan showed multiple bone lesions involving the right femur, ribs, iliac bone, and clavicle. MRI of the femur lesion is shown in Figure 2. Biopsy of the involved femur revealed a small round blue cell tumor with crush artifact (Figure 2). Immunohistochemical studies demonstrated positive CD99, synaptophysin, and NKX2.2. The Ki-67 proliferation index was 80%, and fluorescence in situ hybridization (FISH) analysis showed EWSR1 gene rearrangement. A robotic-assisted laparoscopic radical nephrectomy produced an 8.5 cm lobulated, solid, tan tumor that extended into the pelvic mucosa and perinephric fat. Histologically, the tumor comprised monomorphic small round blue cells with rosettes, necrosis, and a high mitotic count (20 mitoses per 10 high-power fields). The immunoprofile of the renal tumor resembled that of the bone biopsy (Figure 1). Thus, the pathologic stage was pT2, pNx, pM1 (bone), with a histologic grade of 3 (French Federation of Cancer Centers Sarcoma Group). Based on these findings, a diagnosis of renal ESFT was made. Next-generation sequencing (NGS) molecular analysis of the kidney tumor showed a type II* EWSR1:FLI1* gene fusion between exon 7 of *EWSR1* and exon 5 of *FLI1* (Figure 3). The patient experienced a complicated postoperative course and rapid disease progression, including pathologic fractures, sepsis, additional bone metastases, and retroperitoneal lymphadenopathy. After electing hospice care, she expired approximately three months after admission.

Review of the patient’s electronic medical record revealed numerous prior CT scans of the abdomen and pelvis as early as eight years before presentation. Most appeared to be without contrast, presumably for evaluation of her known history of nephrolithiasis. Approximately four years before presentation, she underwent renal ultrasound (RUS), during which a questionable 4.8 cm right lateral midpole mass versus prominent renal tissue was noted, with no further workup per chart review. A chest radiograph in the month before presentation was unremarkable, and a hip radiograph in the days leading up to presentation showed only an 8 mm intertrochanteric cyst in the right femur.

## Discussion

Extraskeletal ESFTs, which represent up to 30% of all cases of ESFT [7], were first described in 1975 by Angervall and Enzinger [8]. In contrast to skeletal ESFT, which most commonly affects males in the second decade of life [9], patients with extraskeletal ESFT are more often female and tend to be older [7,10]. However, current literature suggests that the demographics of patients with renal ESFT seem to match more closely with those of skeletal ESFT [2-5], making this case presentation even rarer. Overall survival of patients with extraskeletal ESFT can be worse in the first two years compared to patients with skeletal ESFT. After two years, patients with skeletal ESFT exhibit lower overall survival [7].

Mor et al. were the first to describe ESFT of renal origin in 1994, at which time peripheral PNETs were already regarded as aggressive neoplasms with early metastasis to other sites such as bone [6]. More recent studies found that nearly half of all patients with renal ESFT had distant metastasis at presentation [2-5]. Bone metastases occur in up to 15% of patients with advanced-stage renal tumors within the first year of diagnosis [11], whereas primary malignant Ewing sarcoma accounts for fewer than 0.2% of all cancers diagnosed in the United States [12].

Symptoms of renal ESFT are nonspecific, initially leading clinicians to consider more common neoplasms [2-4]. Due to the rarity of primary renal ESFT, there is currently no standardized protocol for treating this entity [2-4]. The association between nephrolithiasis and renal ESFT has not yet been elucidated. A 2015 meta-analysis demonstrated an increased risk of other renal cancers in patients with kidney stones, but only in male patients [13].

## Conclusions

While ESFTs are most commonly known to affect the bones, extraskeletal ESFTs have been reported and are often more aggressive. Along with evidence from existing literature, the presence of a sizable renal lesion four years before presentation supports the diagnosis of primary renal ESFT in our patient. This case adds to the body of knowledge on renal ESFTs and confirms their aggressive behavior.
